# Supplementary material for: Inter- and intra-rater reproducibility of quantitative T1 measurement using semiautomatic region of interest placement in myometrium
Source: PLoS One. 2024 Jan 26;19(1):e0297402. doi: 10.1371/journal.pone.0297402 (PMC10817171; doi:10.1371/journal.pone.0297402)
Supplement: S1 Appendix — https://doi.org/10.5281/zenodo.7808462. (DOCX) [file pone.0297402.s001.docx]

**User guide for T1 Map Analysis using MATLAB Code from "Inter- and Intra-rater reproducibility of quantitative T1 measurement using semiautomatic ROI placement in myometrium" Research Paper on Zenodo**

This repository contains the MATLAB code and test data necessary to reproduce the analysis presented in the research. The MATLAB code name is "Generate_T1map_from_VFA_2023_for_submit_b".

All relevant data is included in the manuscript and the MATLAB image analysis code, and an example image that supports the findings of this study is openly available on Zenodo, [***https://doi.org/10.5281/zenodo.7808462***](https://doi.org/10.5281/zenodo.7808462)

- **Prerequisites:**

To run the program, you will need to install the following software installed on your computer:

MATLAB

In addition, you will need to download the MATLAB dataset corresponding to the respective MATLAB program code used in the analysis, as follows:

"Create Generate_T1map_from_VFA_2023_for_submit_b"

Corresponding data: "FA18P_SPGR_DICOM_image" and "FA4P_SPGR_DICOM_image"

- **Procedure:**

To run the MATLAB program, follow these steps:

1. Clone this repository to your local machine.
2. Open a terminal or command prompt and navigate to the directory where the repository is located.
3. Select the corresponding data.
4. Run the program by executing the following command.
5. The program will output the results.
6. ***Detailed descriptions are included at the top of the MATLAB program code.***

- **License:**

This program is not licensed.

- **Contact:**

If you have any questions or comments about the program, please contact:

Sadahiro Nakagawa

Division of Radiology, Asahikawa Medical University Hospital.

2-1-1-1 Midorigaoka-higashi, Asahikawa 078-8510, Japan.

Telephone: +81-166-69-3430

Email: nakasada@asahikawa-med.ac.jp
