# Supplementary material for: Inter- and intra-rater reproducibility of quantitative T1 measurement using semiautomatic region of interest placement in myometrium
Source: PLoS One. 2024 Jan 26;19(1):e0297402. doi: 10.1371/journal.pone.0297402 (PMC10817171; doi:10.1371/journal.pone.0297402)
Supplement: S3 Appendix — https://doi.org/10.5281/zenodo.7807266. (DOCX) [file pone.0297402.s003.docx]

**User guide for converting DICOM to JPEG using MATLAB from the research paper "Inter- and Intra-rater reproducibility of quantitative T1 measurement using semiautomatic ROI placement in myometrium" is possible with the MATLAB procedure and test data provided in the corresponding repository on Zenodo.**

The MATLAB procedure is called "JPEG_image_generate_2023_submit: convert JPEG to DICOM" and includes all the necessary data to reproduce the analysis presented in the research paper. An example image that supports the findings of the study is also available on Zenodo, [***https://doi.org/10.5281/zenodo.7807266***](https://doi.org/10.5281/zenodo.7807266)***.***

Before converting DICOM to JPEG, you need to ensure that MATLAB is installed on your computer. Additionally, you will need to download the MATLAB dataset corresponding to the respective MATLAB program code used in the analysis, which includes "SPRG_DIOM_image" and "IR_DICOM_image".

To convert DICOM to JPEG, follow these steps:

1. Clone this repository to your local machine.
2. Open a terminal or command prompt and navigate to the directory where the repository is located.
3. Run the MATLAB API according to the description provided in the MATLAB program code.
4. Select the corresponding data.
5. The program will output the results.
6. ***Detailed descriptions are included at the top of the MATLAB program code.***

- **Contact:**

If you have any questions or comments about the program, please contact:

Sadahiro Nakagawa

Division of Radiology, Asahikawa Medical University Hospital.

2-1-1-1 Midorigaoka-higashi, Asahikawa 078-8510, Japan.

Telephone: +81-166-69-3430

Email: nakasada@asahikawa-med.ac.jp
