# Supplementary material for: Inter- and intra-rater reproducibility of quantitative T1 measurement using semiautomatic region of interest placement in myometrium
Source: PLoS One. 2024 Jan 26;19(1):e0297402. doi: 10.1371/journal.pone.0297402 (PMC10817171; doi:10.1371/journal.pone.0297402)
Supplement: S4 Appendix — https://doi.org/10.5281/zenodo.7805680. (DOCX) [file pone.0297402.s004.docx]

**User guide for semiautomatic ROI placement analysis using Microsoft Visual Studio 2010 code from "Inter- and Intra-rater reproducibility of quantitative T1 measurement using semiautomatic ROI placement in myometrium" research paper on Zenodo.**

If you're looking to reproduce the semiautomatic ROI placement analysis from the research paper "Inter- and Intra-rater reproducibility of quantitative T1 measurement using semiautomatic ROI placement in the myometrium," you can use the Microsoft Visual Studio 2010 code, which is available in the "***semiautomatic ROI placement 2023 0406***" folder on Zenodo. The folder contains everything you need to replicate the analysis, including an example image that supports the study's findings ([***https://doi.org/10.5281/zenodo.7855051***](https://doi.org/10.5281/zenodo.7855051) ).

To run the code, you'll need to convert your DICOM images to JPEG format using MATLAB, which is available in the "JPEG_image_generate_2023_submit: convert JPEG to DICOM" code folder, also found on Zenodo (DOI: 10.5281/zenodo.7807266; <https://doi.org/10.5281/zenodo.7807266>).

Additionally, you'll need to download the corresponding dataset for the Microsoft Visual Studio 2010 code used in the analysis, which includes the following files: ***"19 18.jpg" (SPGR image)***, ***"19_Z01.jpg" (IR image)***, and ***"T1hosei_map19.xlsx" (Excel sheet of T1map)***.

To perform the semiautomatic ROI placement analysis, please refer to the ***"Semiautomatic ROI placement procedure2023.docx"*** document that outlines the necessary steps using the Microsoft Visual Studio 2010 code provided. To reference the program code, you can unzip the Uterus_MRI.zip folder and click on the Uterus_MRI.sln file in a Microsoft Visual Studio environment.

- **Contact:**

If you have any questions or comments about the program, please contact:

Sadahiro Nakagawa

Division of Radiology, Asahikawa Medical University Hospital.

2-1-1-1 Midorigaoka-higashi, Asahikawa 078-8510, Japan.

Telephone: +81-166-69-3430

Email: nakasada@asahikawa-med.ac.jp
