# Supplementary material for: Inter- and intra-rater reproducibility of quantitative T1 measurement using semiautomatic region of interest placement in myometrium
Source: PLoS One. 2024 Jan 26;19(1):e0297402. doi: 10.1371/journal.pone.0297402 (PMC10817171; doi:10.1371/journal.pone.0297402)
Supplement: Attachment — Submitted filename: Response to Reviewers.docx [file pone.0297402.s009.docx]

Replay to the reviewer and editor

23/April/2023

Dear Sir: Editor-In-Chief

Manuscript reference No. PONE-D-22-17380

Please find attached a revised version of our manuscript “Inter- and Intra-rater reproducibility of quantitative T1 measurement using semiautomatic ROI placement in myometrium”, which we would like to resubmit for publication in PLOS ONE.

Thank you much for your reviews. Your comments were highly insightful and enabled us greatly improve the quality of our manuscript. In the following pages are our point-by-point responses to each of the comments. Furthermore, we attached changed parts of our manuscript. We revised those parts according to the editor’s and reviewer’s comments as 'Response to Reviewers'.

Revisions in the text are shown using yellow highlight for changes and green highlight for deletion. We hope that the revisions in the manuscript are suitable for publication in PLOS ONE.

We shall look forward to hearing from you.

Response to Reviewers

Associate editor:

If you would like to make changes to your financial disclosure, please include your updated statement in your cover letter.

Reply>

Thank you for your kind and informative comments on our manuscript. We greatly appreciate your evaluation of our work. As per your suggestion, we have revised our cover letter to include our updated financial disclosure statement.

Thank you again for your time and consideration. We look forward to hearing from you.

Associate editor:

Guidelines for resubmitting your figure files are available below the reviewer comments at the end of this letter.

Reply>

Thank you for your message regarding resubmitting our figure files (Figure 1-3). We have carefully reviewed the reviewer's comments and have made the necessary revisions to our figures to meet the requirements for publication in your journal. We appreciate the guidelines provided at the end of the letter and have ensured that our revised figures meet the specified criteria. Thank you for your continued guidance and support throughout this process.

Associate editor:

If applicable, we recommend that you deposit your laboratory protocols in protocols.io to enhance the reproducibility of your results. Protocols.io assigns your protocol its own identifier (DOI) so that it can be cited independently in the future. For instructions see: https://journals.plos.org/plosone/s/submission-guidelines#loc-laboratory-protocols. Additionally, PLOS ONE offers an option for publishing peer-reviewed Lab Protocol articles, which describe protocols hosted on protocols.io. Read more information on sharing protocols at <https://plos.org/protocols?utm_medium=editorial-email&utm_source=authorletters&utm_campaign=protocols>.

Reply>

Thank you for your feedback regarding the depositing of our laboratory protocols to Protocols.io. After careful consideration, we have decided that depositing our protocols may not be the most effective way to enhance the reproducibility of our results. However, we understand the importance of reproducibility and have taken steps to ensure that our results can be reproduced.

To enhance the reproducibility of our results, we have provided detailed information on the scan parameters used in our MRI study, as well as the program code for our semiautomatic ROI placement and the procedure for manual ROI placement. We believe that these measures will enable others to reproduce our results and further the field of research.

Thank you for your valuable feedback, and please let us know if you have any further suggestions.

Associate editor:

1. Please ensure that your manuscript meets PLOS ONE's style requirements, including those for file naming. The PLOS ONE style templates can be found at

<https://journals.plos.org/plosone/s/file?id=wjVg/PLOSOne_formatting_sample_main_body.pdf> and

<https://journals.plos.org/plosone/s/file?id=ba62/PLOSOne_formatting_sample_title_authors_affiliations.pdf>

Reply>

Thank you for your message regarding the style requirements for our manuscript. We have consulted the PLOS ONE style templates and made the necessary adjustments to ensure that our manuscript and Title page conform to PLOS ONE's guidelines for formatting and file naming. We appreciate your guidance and assistance in ensuring that our manuscript meets the required standards for publication in your journal.

Associate editor:

2. Please note that PLOS ONE has specific guidelines on code sharing for submissions in which author-generated code underpins the findings in the manuscript. In these cases, all author-generated codes must be made available without restrictions upon publication of the work.

Please review our guidelines at https://journals.plos.org/plosone/s/materials-and-software-sharing#loc-sharing-code and ensure that your code is shared in a way that follows best practices and facilitates reproducibility and reuse.

Reply>

Thank you for bringing to our attention the specific guidelines on code sharing for submissions where author-generated code is fundamental to the manuscript's findings. We have carefully reviewed the guidelines at https://journals.plos.org/plosone/s/materials-and-software-sharing#loc-sharing-code and have ensured that our code is shared in a manner that adheres to best practices, promoting reproducibility and facilitating reuse.

To produce semi-automatic placement of regions of interest (ROIs), we utilized Microsoft Visual Studio 2010 to generate the code. We also developed MATLAB code to generate T1 maps from two SPRG images and created a procedure to convert DICOM files to JPEG format. Additionally, we developed MATLAB code that produces code for the manual placement of ROIs in DICOM format.

In compliance with the guidelines, we have shared the generated code, a test dataset, and the procedure on Zenodo at https://zenodo.org/deposit?page=1&size=20. These resources have been assigned Digital Object Identifiers (DOIs) through Zenodo.

Furthermore, we have included the procedure for replicating our results using these resources in the supporting information (S1-4 Appendix). We have confirmed that these resources are publicly accessible and can be used to replicate the analysis conducted in our study. We hope that other researchers will find these resources valuable in investigating similar research questions.

Thank you for your attention to this matter.

< Materials and Methods P7 line111>

T1 maps were generated pixel by pixel using MATLAB (MathWorks, Natick, MA) with two flip angles (FAs) of 4° and 18° [3]

↓

T1 maps were generated pixel by pixel using MATLAB (MathWorks, Natick, MA) with two flip angles (FAs) of 4° and 18° (S1 Appendix) [3]. The code used to create the T1 map was made available through Zenodo, DOI: [10.5281/zenodo.7808462](https://zenodo.org/badge/DOI/10.5281/zenodo.7808462.svg) (https://doi.org/10.5281/zenodo.7808462).

< Materials and Methods P8 line128>

Manual ROI placement was performed using MATLAB. First, a whole uterus ROI was manually placed to trace the external contour of the myometrium (Fig.1a).

↓

Manual ROI placement was performed using MATLAB~~. First, a whole uterus ROI was manually placed to trace the external contour of the myometrium (Fig.1a).~~ (S2 Appendix) using the following procedure: (1) The T1 map data was transferred to the MATLAB workspace; (2) An IR image was selected by the observer in the MATLAB application to place the ROI; (3) The provided code was executed to open the image segmentation application in MATLAB; (4) A whole uterus ROI was manually placed to trace the external contour of the myometrium; (5) An endometrial ROI was manually placed to trace the external contour of the endometrium; (6) The myometrial ROI was created by subtracting the endometrial ROI from the whole uterus ROI; (7) Mean qT1 values were calculated from the corresponding pixel values of the T1 map within the myometrial ROI. The code for manual ROI placement was made available through Zenodo, DOI: [DOI/10.5281/zenodo.7811343](https://zenodo.org/badge/DOI/10.5281/zenodo.7811343.svg) (https://doi.org/10.5281/zenodo.7811343).

< Materials and Methods P9 line144>

We developed an in-house software using Microsoft Visual Studio 2010 (Microsoft Corporation, Redmond, WA, USA) to read and display digital imaging and communications in medicine (DICOM) files on a personal computer with Windows 10, 64-bit operating system (Microsoft). The myometrial ROI was placed using the following semiautomatic procedure (Fig 1d-f): (1) the center of the endometrium was determined by the user; (2) the user traced the external contour of the myometrium (Fig 1d); (3) the endometrial ROI was automatically extracted by the software using a signal intensity threshold (Fig 1e), which was initially set at within ±3% of the endometrial signal intensity determined by the user and was increased in increments of 1% to maximize the area of the endometrial ROI, while excluding the external contour of the myometrium; (4) pixel values were sampled from the area between the endometrial ROI and external contour of the myometrium; (5) the mean and standard deviation of the sampled pixel values were calculated; (6) the myometrial ROI was extracted automatically based on pixels that were within ±1.96 standard deviations of the mean signal intensity; (7) the myometrial ROI was placed on the SPGR image; (8) we calculated the mean pixel value of the myometrial ROI; (9) the sum of squared deviation was calculated with variation of ROI locations, and the vertical and horizontal coordinates were changed from −10 to +10 pixels; (10) the optimal combination of vertical and horizontal coordinates with minimum sum of squared deviation was obtained, and the appropriateness of the ROI location was visually confirmed by experts; and (11) the mean qT1 value was calculated from the corresponding pixel values of the T1 map within the optimally placed myometrial ROI (Fig 1f). Eventually, the required operations by the user were only 2 steps: (1) click center of endometrium, (2) roughly draw the shape of myometrium manually.

↓

We developed an in-house software using Microsoft Visual Studio 2010 (Microsoft Corporation, Redmond, WA, USA) to read and display digital imaging and communications in medicine (DICOM) files on a personal computer with Windows 10, a 64-bit operating system (Microsoft). Prior to analysis, we converted the DICOM images to JPEG format using MATLAB (S3 Appendix). The source code for the in-house software used to read and display DICOM files, as well as the MATLAB code used to convert DICOM images to JPEG format, has been made available through Zenodo, DOI: 10.5281/zenodo.7807266 (<https://doi.org/10.5281/zenodo.7807266>).

The myometrial ROI was placed using the following semiautomatic procedure (Fig 1d-f, S4 Appendix): (1) the center of the endometrium was determined by the user; (2) the user traced the external contour of the myometrium (Fig 1d); (3) the endometrial ROI was automatically extracted by the software using a signal intensity threshold (Fig 1e), which was initially set at within ±3% of the endometrial signal intensity determined by the user and was increased in increments of 1% to maximize the area of the endometrial ROI, while excluding the external contour of the myometrium; (4) pixel values were sampled from the area between the endometrial ROI and external contour of the myometrium; (5) the mean and standard deviation of the sampled pixel values were calculated; (6) the myometrial ROI was extracted automatically based on pixels that were within ±1.96 standard deviations of the mean signal intensity; (7) the myometrial ROI was placed on the SPGR image; (8) we calculated the mean pixel value of the myometrial ROI; (9) the sum of squared deviation was calculated with variation of ROI locations, and the vertical and horizontal coordinates were changed from −10 to +10 pixels; (10) the optimal combination of vertical and horizontal coordinates with minimum sum of squared deviation was obtained, and the appropriateness of the ROI location was visually confirmed by experts; and (11) the mean qT1 value was calculated from the corresponding pixel values of the T1 map within the optimally placed myometrial ROI (Fig 1f). Eventually, the required operations by the user were only 2 steps: (1) click the center of the endometrium, (2) roughly draw the shape of the myometrium manually. Our algorithm did not use any active contour model for ROI extraction. The Code for semiautomatic ROI placement was made available through the Zenodo, DOI: DOI/10.5281/zenodo.7855051 (<https://doi.org/10.5281/zenodo.7855051>).

Associate editor:

3. Thank you for stating the following financial disclosure:

"Atsutaka Okizaki received Research funding of Nihon Medi-physics Co. and FUJIFILM Toyama Chemical Co., Ltd. The other authors have no conflict of interest."

Please state what role the funders took in the study. If the funders had no role, please state: "The funders had no role in study design, data collection and analysis, decision to publish, or preparation of the manuscript." If this statement is not correct you must amend it as needed.

Please include this amended Role of Funder statement in your cover letter; we will change the online submission form on your behalf.

Replay>

Thank you very much for providing us with such detailed feedback. We truly appreciate it. We want to let you know that we have carefully considered your comments, and as the funders did not play a role in this study, we have revised our statement on competing interests accordingly. The updated statement now reads as follows: "The funders had no involvement in the study design, data collection and analysis, decision to publish, or preparation of the manuscript." We have made sure to include this revised statement in our cover letter.

Associate editor:

4. Thank you for stating the following in the Financial Disclosure section:

We note that you received funding from a commercial source: Nihon Medi-physics Co. and FUJIFILM Toyama Chemical Co., Ltd.

Please provide an amended Competing Interests Statement that explicitly states this commercial funder, along with any other relevant declarations relating to employment, consultancy, patents, products in development, marketed products, etc.

Within this Competing Interests Statement, please confirm that this does not alter your adherence to all PLOS ONE policies on sharing data and materials by including the following statement: “This does not alter our adherence to PLOS ONE policies on sharing data and materials.” (As detailed online in our guide for authors http://journals.plos.org/plosone/s/competing-interests).

If there are restrictions on sharing of data and/or materials, please state these. Please note that we cannot proceed with consideration of your article until this information has been declared.

Please include your amended Competing Interests Statement within your cover letter. We will change the online submission form on your behalf.

Replay>

Thank you very much for your valuable feedback. We would like to provide an amended Competing Interests Statement that explicitly states the commercial funders, Nihon Medi-physics Co. and FUJIFILM Toyama Chemical Co., Ltd.: “This does not alter our adherence to PLOS ONE policy on sharing data and materials”.

We confirmed that this statement does not affect our adherence to all PLOS ONE policies on sharing data and materials. Please let us know if any further amendments are necessary.

Associate editor:

5. In your Data Availability statement, you have not specified where the minimal data set underlying the results described in your manuscript can be found. PLOS defines a study's minimal data set as the underlying data used to reach the conclusions drawn in the manuscript and any additional data required to replicate the reported study findings in their entirety. All PLOS journals require that the minimal data set be made fully available. For more information about our data policy, please see http://journals.plos.org/plosone/s/data-availability.

Upon re-submitting your revised manuscript, please upload your study’s minimal underlying data set as either Supporting Information files or to a stable, public repository and include the relevant URLs, DOIs, or accession numbers within your revised cover letter. For a list of acceptable repositories, please see http://journals.plos.org/plosone/s/data-availability#loc-recommended-repositories. Any potentially identifying patient information must be fully anonymized.

Important: If there are ethical or legal restrictions to sharing your data publicly, please explain these restrictions in detail. Please see our guidelines for more information on what we consider unacceptable restrictions to publicly sharing data: http://journals.plos.org/plosone/s/data-availability#loc-unacceptable-data-access-restrictions. Note that it is not acceptable for the authors to be the sole named individuals responsible for ensuring data access.

We will update your Data Availability statement to reflect the information you provide in your cover letter.

Replay>

Thank you very much for your helpful and informative feedback. The minimal dataset underlying the results described in this study, including the inter- and intra-observer reliability of qT1 evaluation times obtained by observers 1 and 2 using manual and semiautomatic ROI placements, is provided in the manuscript and its Supporting Information files (S5–8 Files). We have created a Data Availability Statement and included it in our cover letter, which reads as follows:

Data Availability Statement:

The minimal dataset underlying the results described in this study are within the manuscript and its Supporting Information files. The source code analysis of Microsoft Visual Studio 2010 image analysis code and MATLAB, along with an example image that supports the findings of this study, has been openly published on Zenodo at [https://zenodo.org/deposit?page=1&size=20]. Additionally, any other data required to fully replicate the reported study findings can be obtained by contacting the corresponding author upon reasonable request.

Thank you for your guidance in ensuring our manuscript meets PLOS data policy requirements. Please let us know if any further revisions or clarifications are needed.

Associate editor:

6. PLOS requires an ORCID iD for the corresponding author in Editorial Manager on papers submitted after December 6th, 2016. Please ensure that you have an ORCID iD and that it is validated in Editorial Manager. To do this, go to ‘Update my Information’ (in the upper left-hand corner of the main menu), and click on the Fetch/Validate link next to the ORCID field. This will take you to the ORCID site and allow you to create a new iD or authenticate a pre-existing iD in Editorial Manager. Please see the following video for instructions on linking an ORCID iD to your Editorial Manager account: https://www.youtube.com/watch?v=_xcclfuvtxQ

Replay>

Thank you very much in advance for your kind support.

We have provided the ORCID iD for the corresponding author, Atsutaka Okizaki, below:

ORCID iD: [0000-0001-8203-827X]

Associate editor:

7. Please upload a new copy of Figure 1 as the detail is not clear. Please follow the link for more information:

https://blogs.plos.org/plos/2019/06/looking-good-tips-for-creating-your-plos-figures-graphics/

https://blogs.plos.org/plos/2019/06/looking-good-tips-for-creating-your-plos-figures-graphics/

Replay>

Thank you very much for your helpful and informative comment. We have corrected the resolution of Figure 1 based on your comment and have attached a clearer version of Figure 1 for your convenience.

Reviewer #1:

1. Manual ROI placement was performed using MATLAB: Please specify which method was used.

Reply>

Thank you for your kind and informative comments on our manuscript. We have developed MATLAB code that generates code for the manual placement of ROIs in DICOM format. To comply with the guidelines, we have shared the generated code, a test dataset, and the procedure on Zenodo at DOI: 10.5281/zenodo.7811343. The resource has been assigned DOI through Zenodo. Additionally, we have included the procedure for replicating our results using these resources in the supporting information (S2 Appendix).

< Materials and Methods P8 line128>

Manual ROI placement was performed using MATLAB. First, a whole uterus ROI was manually placed to trace the external contour of the myometrium (Fig.1a).

↓

Manual ROI placement was performed using MATLAB~~. First, a whole uterus ROI was manually placed to trace the external contour of the myometrium (Fig.1a).~~ (S2 Appendix) using the following procedure: (1) The T1 map data was transferred to the MATLAB workspace; (2) An IR image was selected by the observer in the MATLAB application to place the ROI; (3) The provided code was executed to open the image segmentation application in MATLAB; (4) A whole uterus ROI was manually placed to trace the external contour of the myometrium; (5) An endometrial ROI was manually placed to trace the external contour of the endometrium; (6) The myometrial ROI was created by subtracting the endometrial ROI from the whole uterus ROI; (7) Mean qT1 values were calculated from the corresponding pixel values of the T1 map within the myometrial ROI. The code for manual ROI placement was made available through Zenodo, DOI: [DOI/10.5281/zenodo.7811343](https://zenodo.org/badge/DOI/10.5281/zenodo.7811343.svg) (https://doi.org/10.5281/zenodo.7811343).

Reviewer #1:

2. In-house software using Microsoft Visual Studio 2010: many semi automatic segmentation models are there, please specify which algorithm was employed in this research work, upon observation of outputs, seems to have developed an active contour model for ROI extraction, if so, please specify in detail about that.

Replay>

Thank you for bringing this to our attention. Our algorithm does not utilize any active contour model for ROI extraction. Instead, we fully developed and programmed a semi-automatic ROI placement approach from scratch. The program code has been published on Zenodo: DOI: 10.5281/zenodo.78550551.

< Materials and Methods P9 line160>

Eventually, the required operations by the user were only 2 steps: (1) click center of endometrium, (2) roughly draw the shape of myometrium manually.

↓

Eventually, the required operations by the user were only 2 steps: (1) click center of endometrium, (2) roughly draw the shape of myometrium manually. Our algorithm did not use any active contour model for ROI extraction.

Reviewer #1:

3. Any preprocessing was done prior to ROI placement

Replay>

Thank you for your message. I'm glad to have been able to assist you.

To further clarify, prior to running the semiautomatic analysis, the authors developed a MATLAB code (available on Zenodo, DOI: 10.5281/zenodo.7808462) for generating T1 maps from two SPRG images. Additionally, MATLAB (available on Zenodo, DOI: 10.5281/zenodo.7807266) was used by the authors to convert the DICOM images into JPEG format, with automatic optimization of brightness during the conversion process.

Reviewer #1:

4. Quantitative T1 (qT1) values with variable flip angles (VFA) are important for characterizing lesions in 24 patients with pelvic disease: whether this is applicable only for pelvic disease diagnosis

Replay>

Thank you for your inquiry regarding the applicability of our software beyond the uterus. While our software was originally designed for the analysis of the uterus, we believe that its programmatic design may potentially allow for its use in analyzing other organs as well, including the pelvic region. However, we acknowledge that further research is necessary to confirm this hypothesis. We are committed to continuing our investigation and development of this software to explore its potential for expanding its applications.

< Discussion P14 line234>

The algorithm for semiautomatic ROI placement provided high reproducibility. In principle, the algorithm can be applied to other medical imaging modalities for delineating the uterus because it uses pixel values that correspond with anatomical structures.

↓

The algorithm for semiautomatic ROI placement provided high reproducibility. In principle, the algorithm can be applied to other medical imaging modalities for delineating the uterus because it uses pixel values that correspond with anatomical structures. Although this software was specifically designed for the analysis of the uterus, we believe that its programmatic design theoretically allows for its use in analyzing other organs as well. With further research, it may be possible to extend the use of this software beyond its current scope. Therefore, we plan to continue investigating and developing this software to explore its potential for expanding applications.

Reviewer #1:

5. Please improve the quality of figure 3

Replay>

Thank you for your message. We greatly appreciate your helpful and informative comment. As per your suggestion, we have corrected the resolution of Figure 3 and have also attached a clearer version of Figure 3 for your convenience.
