# Supplementary material for: Inter- and intra-rater reproducibility of quantitative T1 measurement using semiautomatic region of interest placement in myometrium
Source: PLoS One. 2024 Jan 26;19(1):e0297402. doi: 10.1371/journal.pone.0297402 (PMC10817171; doi:10.1371/journal.pone.0297402)
Supplement: Attachment — Submitted filename: Response to Reviewers.docx [file pone.0297402.s010.docx]

Replay to the reviewer and editor

30/December/2023

Dear Sir: Editor-In-Chief

Manuscript reference No. PONE-D-22-17380R1

We are pleased to resubmit our revised manuscript, “Inter- and intra-rater reproducibility of quantitative T1 measurement using semiautomatic region of interest placement in myometrium”, for your consideration in PLOS ONE.

Thank you very much for your reviews. Your comments were highly insightful and enabled us to improve the quality of our manuscript greatly. In the following pages are our point-by-point responses to each of the comments. Furthermore, we attached changed parts of our manuscript. We revised those parts according to the editor’s and reviewer’s comments as ‘Response to Reviewers'.

Revisions in the text are shown using yellow highlight for changes and green highlight for deletion. We hope that the revisions in the manuscript are suitable for publication in PLOS ONE.

We shall look forward to hearing from you.

**Response to Reviewers**

**Associate editor:**

**If applicable, we recommend that you deposit your laboratory protocols in protocols.io to enhance the reproducibility of your results. Protocols.io assigns your protocol its own identifier (DOI) so that it can be cited independently in the future. For instructions see: https://journals.plos.org/plosone/s/submission-guidelines#loc-laboratory-protocols. Additionally, PLOS ONE offers an option for publishing peer-reviewed Lab Protocol articles, which describe protocols hosted on protocols.io. Read more information on sharing protocols at** [**https://plos.org/protocols?utm_medium=editorial-email&utm_source=authorletters&utm_campaign=protocols**](https://plos.org/protocols?utm_medium=editorial-email&utm_source=authorletters&utm_campaign=protocols).

Reply>

Thank you for your kind and informative comments on our manuscript. We greatly appreciate your evaluation of our work. As per your suggestion, we submitted our laboratory protocols for qT1 measurements to protocols.io, aiming to improve the reproducibility of our results. The uploaded files were S1-4 Appendix of program code for qT1 measurements.

T1 maps were generated pixel by pixel using MATLAB (MathWorks, Natick, MA) with two flip angles (FAs) of 4° and 18° (S1 Appendix). Manual ROI placement was performed using MATLAB (S2 Appendix). Before analysis, we converted the DICOM images to JPEG format using MATLAB (S3 Appendix). The myometrial ROI was placed using the following semiautomatic procedure (S4 Appendix).

Thank you again for your time and consideration. We look forward to hearing from you.

**Associate editor:**

**Please review your reference list to ensure that it is complete and correct. If you have cited papers that have been retracted, please include the rationale for doing so in the manuscript text, or remove these references and replace them with relevant current references. Any changes to the reference list should be mentioned in the rebuttal letter that accompanies your revised manuscript. If you need to cite a retracted article, indicate the article’s retracted status in the References list and also include a citation and full reference for the retraction notice.**

Reply>

We have carefully reviewed the reviewer's comments and made the necessary revisions to our manuscript. In response to your specific request, we have revised the reference list as follows:

The deleted reference numbers are marked in green:

7, 8, 15, 18, 21, 22

The changed reference numbers are marked in yellow:

9 →　7

10 →　8

11 →　9

12→　10

13 →　11

14 →　12

16→　13

17 →　14

19 →　15

20 →　16

23 →　17

24 →　18

25 →　19

26 →　20

**Associate editor:**

**While revising your submission, please upload your figure files to the Preflight Analysis and Conversion Engine (PACE) digital diagnostic tool, https://pacev2.apexcovantage.com/. PACE helps ensure that figures meet PLOS requirements. To use PACE, you must first register as a user. Registration is free. Then, login and navigate to the UPLOAD tab, where you will find detailed instructions on how to use the tool. If you encounter any issues or have any questions when using PACE, please email PLOS at figures@plos.org. Please note that Supporting Information files do not need this step.**

Reply>

We have resubmitted our figure files that were uploaded to the PACE digital diagnostic tool and downloaded from PACE.

**Reviewer #2:**

**1. The abstract should be rewritten to provide essential information to the readers. The purpose heading is quite long, providing some unnecessary information, while the material and methods one is quite short, without any information regarding the statistics analysis. Be sure to define all abbreviations (e.g. IR). Purpose and conclusions should be aligned, in the conclusion heading manual segmentation is not mentioned at all.**

Reply>

Thank you for your message regarding resubmitting our abstract. In response to your specific comments, we have revised the purpose to eliminate unnecessary details, and we have expanded the material and methods section to include information about the statistical analysis. Furthermore, we have ensured that all abbreviations, including "IR," are explicitly defined within the abstract. Furthermore, we added the description for the manual ROI placement in the conclusion.

< Abstract P2 line45>

Purpose: Quantitative T1 (qT1) values with variable flip angles (VFA) are important for characterizing lesions in patients with pelvic disease. QT1 values vary depending on the location, shape, and area of the region of interest (ROI) when the ROI is set manually.

The objective of this study was to investigate the inter- and intraobserver reproducibility of qT1 measurements using manual and semiautomatic ROI settings. We hypothesized that the semiautomatic method, which uses a 3D anatomical relationship between the myometrium and other tissues, may be useful for minimizing the variation in ROI settings and thus, improve qT1 reproducibility.

↓

Purpose: Quantitative T1 (qT1) values with variable flip angles (VFA) are important for characterizing lesions in patients with pelvic disease. QT1 values vary depending on the location, shape, and area of the region of interest (ROI) when the ROI is set manually.

The objective of this This study was aimed to investigate the inter- and intraobserver reproducibility of quantitative T1 (qT1) measurements using manual and semiautomatic region of interest (ROI) settings placements. We hypothesized that the usefulness of the semiautomatic method, which uses utilizes a 3D three-dimensional (3D) anatomical relationship between the myometrium and other tissues, may be useful for minimizing the ROI placement variation, thereby improving in ROI settings and thus, improve qT1 reproducibility compared to the manual approach. The semiautomatic approach, which considered anatomical relationships, was expected to enhance reproducibility by reducing ROI placement variabilities.

< Abstract P2 line52>

Materials and methods: Twenty-three healthy female volunteers were investigated. The data with VFA and IR were acquired using 3D-spoiled gradient echo and spin echo sequences, respectively. T1 maps were generated with VFA. Manual and semiautomatic ROI placements were performed independently. Mean qT1 values were calculated from the T1 maps using the corresponding pixel values of the myometrial ROI. Inter- and intraobserver reproducibility of qT1 values were assessed.

↓

Materials and methods: Twenty-three This study recruited 23 healthy female volunteers were investigated. The data. Data with variable flip angle (VFA) and IR inversion recovery were acquired using 3D-spoiled gradient echo and spin echo sequences, respectively. T1 maps were generated with VFA. Manual and semiautomatic ROI placements were performed independently conducted. Mean qT1 values were calculated from the T1 maps using the corresponding pixel values of the myometrial ROI. Inter- and intraobserver reproducibility of qT1 values were assessed was investigated. The inter- and intraobserver reproducibility of qT1 values was evaluated by calculating the coefficient of variation (CoV). Further, reproducibility was evaluated with inter- and intraobserver errors and intraclass correlation coefficients (ICCs). Bland–Altman analysis was utilized to compare the results, estimate bias, and determine the limits of agreement.

< Abstract P2 line57>

The mean inter- and intraobserver error for semiautomatic ROI placement were also significantly lower than those for manual ROI placement (p < 0.05 and p < 0.01, respectively).

↓

The Further, the mean inter- and intraobserver error errors for semiautomatic ROI placement were also significantly lower than those for manual ROI placement (p < 0.05 and p < 0.01, respectively).

< Abstract P2 line62>

Conclusion: Semiautomatic ROI placement has high reproducibility of qT1 measurements.

↓

Conclusion: Semiautomatic ROI placement has demonstrated high reproducibility of qT1 measurements compared with manual methods. Semiautomatic ROI placement may be useful for evaluating uterine qT1 with high reproducibility.

**Reviewer #2:**

**2. Introduction: The manuscript requires a careful proof-reading, preferably by an English native speaker, many errors and typos (Line 80, pervious) are present, especially in this heading.**

Replay>

Our revised manuscript was edited by a native English speaker through a new editing process.

We have deleted this sentence concluding type error (Line 80, pervious).

The manuscript has undergone additional English proofreading, and we sent a new editorial certificate. We have reviewed and revised the title through English proofreading.

< Introduction P4 line80>

Pervious paper showed peak of qT1 in the uterine fibroid was markedly decreased after the therapy, although that in the myometrium did not lead to a large change [15]

↓

deleted

< Title >

Inter- and Intra-rater reproducibility of quantitative T1 measurement using semiautomatic ROI placement in myometrium

↓

Inter- and Intra intra-rater reproducibility of quantitative T1 measurement using semiautomatic ROI region of interest placement in myometrium

**Reviewer #2:**

**Some relevant references are missing (e.g. Line 70, Line 90).**

Replay>

Thank you for your message. We have added relevant references to the sentence in Line 70, which removed the text including the relevant references to the sentence in Line 90.

< Introduction P4 line69>

Quantitative image analysis has the advantage of better tissue identification and classification compared with visual assessment of traditional T1- and T2-weighted images.

↓

Quantitative image analysis has the advantage of better tissue identification and classification compared with the visual assessment of traditional T1- and T2-weighted images [4-6].

< Introduction P4 line89>

Of these pulse sequences, IR is most frequently used as the gold standard for qT1 evaluation.

↓

deleted

**Reviewer #2:**

**Please define all the abbreviations (e.g. UAE, IR).**

Replay>

We have defined the abbreviations for the words that were pointed out. We have checked all the abbreviations.

<Introduction P5 line88>

Various pulse sequences, including IR [17], modified look-locker inversion recovery (MOLLI) [2], and VFA [18], have been used for measuring qT1.

↓

deleted

We have added the below text in the ‘Subjects’ of the 'Materials and Methods' section.

< Materials and Methods P6 line114>

none

↓

This study excluded subjects who met the following exclusion criteria: (a) subjects who presented with uterine mass lesions (n = 5), (b) subjects who had misregister between inversion recovery (IR) and each VFA image (n = 13), and (c) subjects who observed motion artifacts in the spoiled gradient recalled echo (SPGR) image acquired for qT1 with VFA (n = 15). Our study enrolled 23 healthy women volunteers (age range: 22–51 years; mean age ± standard deviation [SD]: 33 ± 11 years).

<Introduction P4 line78>

Pervious paper showed peak of qT1 in the uterine fibroid was markedly decreased after the therapy, although that in the myometrium did not lead to a large change [15]. The assessment of myometrium may desire for the high intensity focused ultrasound treatment.

↓

Pervious paper showed peak of qT1 in the uterine fibroid was markedly decreased after the therapy, although that in the myometrium did not lead to a large change [15]. The assessment of myometrium may desire for the high intensity focused ultrasound treatment.

Dominant leiomyoma qT1 measures were used to predict uterine and dominant leiomyoma size reductions after uterine artery embolization (UAE) [12].

**Reviewer #2:**

**Lines 81-82 Please rephrase**

Replay>

We greatly appreciate your helpful and informative comment. As per your suggestion, we have deleted this text.

<Introduction P4 line79>

The assessment of myometrium may desire for the high intensity focused ultrasound treatment.

↓

deleted

**Reviewer #2:**

**Line 105, What does “continuity” means in this setting?**

Replay>

As per your suggestion, we have corrected the text.

<Introduction P6 line103>

We hypothesized that a semiautomatic method that uses a 3D anatomical relationship between the myometrium and endometrium and continuity of MRI may be useful to minimize variation and improve the reproducibility of the ROI settings.

↓

We hypothesized that a semiautomatic method that uses, using a 3D anatomical relationship between the myometrium and endometrium and continuity of MRI, may be useful to minimize for minimizing variation and improve the by manual ROI placements and improving ROI placement reproducibility of the ROI settings.

**Reviewer #2:**

**3.** **Provide a clear** **statement regarding study nature**

Replay>

We have extensively revised the introduction to provide a clear statement regarding the nature of our study.

<Introduction P6 line103>

A highly reproducible ROI placement without the need for a contrast agent is preferable for quantitative MRI. We hypothesized that a semiautomatic method that uses a 3D anatomical relationship between the myometrium and endometrium and continuity of MRI may be useful to minimize variation and improve the reproducibility of the ROI settings. We developed a semiautomatic myometrial ROI placement method that requires no contrast agent. The objective of this study is to investigate the inter- and intraobserver reproducibility of qT1 measurements using manual and semiautomatic ROI settings.

↓

A highly Highly reproducible ROI placement without the need for a contrast agent is preferable for quantitative MRI.

Therefore, we developed a semiautomatic myometrial ROI placement method without a contrast agent. We hypothesized that a semiautomatic method that uses, using a 3D anatomical relationship between the myometrium and endometrium and continuity of MRI, may be useful to minimize for minimizing variation and improve the by manual ROI placements and improving ROI placement reproducibility of the ROI settings. We developed a semiautomatic myometrial ROI placement method that requires no contrast agent. The objective of this. This study is aimed to investigate the inter- and intraobserver reproducibility of qT1 measurements using manual and semiautomatic ROI settings placements.

**Reviewer #2:**

**4. Line 148, “There were no adequate”. Maybe is it “inadequate”? Otherwise I’m not getting what this sentence mean.**

Replay>

As per your suggestion, we have removed the text including the sentence.

< Materials and Methods P4 line148>

There were no adequate image quality and image misregistration between IR image and 2 SPGRs on visual assessment.

↓

There were no adequate, evaluated the MR images using IR images and two SPGRs to confirm image quality and image misregistration between IR image registration.

**Reviewer #2:**

**5. Results: Consider to add more results (and less cross-reference to tables or figures) and p-values in this sections.**

Replay>

As per your suggestion, we have added more results and p-values in this section.

< Materials and Methods P24 line252>

A negative proportional bias was observed between the first and second measurements of qT1 using manual ROI placement (Fig.3c). However, no proportional bias was observed using semiautomatic ROI placement (Fig.3d).

The means and standard deviations of time with manual and semiautomatic ROI placements were 55±4 sec, 44±5 sec, respectively. The times with semiautomatic ROI placement were significant smaller than those with manual ROI placement (P < 0.001).

↓

Positive proportional bias emerged in the qT1 measurements between observers 1 and 2 using semiautomatic ROI placement (R = 0.461, *p* = 0.027; Fig. 3b). A negative proportional bias was observed determined between the first and second qT1 measurements of qT1 using manual ROI placement (R = 0.532, *p* = 0.009; Fig. 3c). However, no proportional bias was observed using semiautomatic ROI placement (Fig.in the measurements between observers 1 and 2 using manual ROI placement (R = 0.224, *p* = 0.304; Fig. 3a). Similarly, no proportional bias was observed between the first and second measurements using semiautomatic ROI placement (R = 0.11, *p* = 0.959; Fig. 3d).

The means and standard deviations SDs of time with manual and semiautomatic ROI placements were 55 ± 4 sec s and 44 ± 5 sec s, respectively. The times with semiautomatic ROI placement were significant significantly smaller than those with manual ROI placement (P*p* < 0.001).

**Reviewer #2:**

**6. How many patients were excluded due to the presence of mass lesions? It is stated in the methods the presence of this exclusion criterion.**

Replay>

We have added the exclusion criteria in the ‘Subjects” section.

< Materials and Methods P6 line112>

Subjects

The study protocol was approved by our institutional research ethics committee at Asahikawa Medical University in accordance with the principles of the Declaration of Helsinki. All subjects were informed regarding the content of the study and were provided written informed consent. Between May 2018 and April 2019, we enrolled 23 healthy women volunteers (age range, 22–51 years; mean age ± standard deviation, 33 ± 11 years) in our study.

↓

The study protocol was approved by our Our institutional research ethics committee at Asahikawa Medical University in accordance with approved the study protocol following the principles of the Declaration of Helsinki. All subjects were informed regarding the content of the study and were provided signed written informed consent. Between From May 2018 and to April 2019, we enrolled 23 healthy women volunteers (age range, 22–51 years; mean age ± standard deviation, 33 ± 11 years)56 subjects underwent pelvic MR imaging, incorporating VFA for qT1 measurements in our study. hospital.

This study excluded subjects who met the following exclusion criteria: (a) subjects who presented with uterine mass lesions (n = 5), (b) subjects who had misregister between inversion recovery (IR) and each VFA image (n = 13), and (c) subjects who observed motion artifacts in the spoiled gradient recalled echo (SPGR) image acquired for qT1 with VFA (n = 15). Our study enrolled 23 healthy women volunteers (age range: 22–51 years; mean age ± standard deviation [SD]: 33 ± 11 years).

**Reviewer #2:**

**7 Discussion, especially after the particularly long introduction, sounds a bit redundant. Consider to revise both these sections to avoid redundancy, trying to focus the discussion on drawing conclusions regarding the comparison between manual and semiautomatic segmentation.**

Replay>

As per your suggestion, we have extensively revised the introduction in response to the reviewer's comments. Furthermore, we have added a comparison of manual and semiautomatic ROI placement in the conclusion.

<Introduction P6 line103>

Quantitative image analysis of magnetic resonance imaging (MRI) data may be useful for comparing subjects. Quantitative T1 (qT1), T2, and apparent diffusion coefficient values have been used to investigate various diseases [1-4]. Quantitative image analysis has the advantage of better tissue identification and classification compared with visual assessment of traditional T1- and T2-weighted images. QT1 values have been used extensively to study the central nervous system over the past several decades [5-7]. Visualizing qT1 maps are more useful for identifying deep cerebellar nuclei than traditional T1-weighted images [6]. Recently, qT1 values have also been used in the diagnoses of heart disease, where the detection rates of acute myocardial edema with qT1 were higher than those with T2-weighted images[8]. Quantitative analyses are applicable for diagnoses not only in the central nervous system and myocardium but also in the pelvis [9-12].

Davenport et al. reported that qT1 using dynamic contrast-enhanced MRI with variable flip angle (VFA) in patients with uterine myoma was important for characterizing the lesion and monitoring drug effects [13]. QT1 in myometrium was also available for investigating uterine pathology. The preinterventional qT1 of leiomyoma and uterus also could predict the ability of UAE to reduce the sizes of the leiomyoma and the histological change of uterus [14]. Pervious paper showed peak of qT1 in the uterine fibroid was markedly decreased after the therapy, although that in the myometrium did not lead to a large change [15]. The assessment of myometrium may desire for the high intensity focused ultrasound treatment.

QT1 values vary depending on various factors, such as magnetic field strength, magnet manufacturer, imaging sequence, image acquisition policy, postprocessing algorithm, body size and age of patients, and measurement method of quantitative values [16, 17]. Therefore, quantitative values are not directly comparable between different institutions without the use of certain reference values, and each institution needs to define reference values for standardization. In principle, such reference values would require high reproducibility of measurements.

Various pulse sequences, including IR[17], modified look-locker inversion recovery (MOLLI)[2], and VFA[18], have been used for measuring qT1. Of these pulse sequences, IR is most frequently used as the gold standard for qT1 evaluation. However, acquisition with IR requires considerable time (from 30 minutes to several hours). Acquisitions with MOLLI and VFA are shorter compared with IR, although MOLLI has the disadvantage of data of only one slice being obtained per acquisition, and thus, acquiring many slices is still time-consuming. By contrast, the VFA method requires a shorter acquisition time because multiple slices are acquired per acquisition.

A robust, reproducible, and standardized method is desirable for quantitative measurements. QT1 values sometimes vary, which can depend on location, shape, and area of region of interest (ROI) when the ROI is set manually. Heye et al. reported that a semiautomatic ROI placement improved the reproducibility of pharmacokinetic parameters, but the method is only available using dynamic enhanced MRI [19]. Chapiro et al. highlighted the usefulness of semiautomated 3D tumor segmentation but noted the necessity of acquiring contrast-enhanced T1-weighted images [20].

Gadolinium contrast agents are commonly used in clinical practice. However, they have been known to sometimes cause side effects, making them unsuitable for patients with severe kidney dysfunction due to avoid nephrogenic systemic fibrosis [21, 22]. Therefore, contrast agents should only be used if necessary.

↓

Quantitative image analysis of magnetic Magnetic resonance imaging (MRI) data quantitative image analysis may be useful for comparing subjects. Quantitative T1 (qT1), T2, and apparent diffusion coefficient values have been used utilized to investigate various diseases [1-4]. Quantitative image analysis has the advantage of better tissue identification and classification compared with the visual assessment of traditional T1- and T2-weighted images [4-6]. QT1 values have been used extensively used to study the central nervous system over the past several decades [5-7]. Visualizing qT1 maps are more useful for identifying deep cerebellar nuclei than traditional T1-weighted images [6]. Recently, qT1 values have also been used in the diagnoses of heart disease, where the detection rates of acute myocardial edema with qT1 were higher than those with T2-weighted images[8]. Quantitative analyses are applicable for diagnoses not only in the central nervous system and myocardium but also in the pelvis[9-12] [7-10].

Davenport et al. reported that revealed qT1, using dynamic contrast-enhanced MRI with variable flip angle (VFA) in patients with uterine myoma was important, to be crucial for characterizing the lesion and monitoring drug effects [1311]. QT1 in myometrium was also available for investigating uterine pathology. The preinterventional qT1 of leiomyoma and uterus also could predict the ability of UAE to reduce the sizes of the leiomyoma and the histological change of uterus[14]. Pervious paper showed peak of qT1 in the uterine fibroid was markedly decreased after the therapy, although that in the myometrium did not lead to a large change[15]. The assessment of myometrium may desire for the high intensity focused ultrasound treatment.

. Dominant leiomyoma qT1 measures were used to predict uterine and dominant leiomyoma size reductions after uterine artery embolization (UAE) [12].

QT1 values vary depending based on various factors, such as including magnetic field strength, magnet manufacturer, imaging sequence, image acquisition policy, postprocessing algorithm, body size and age of patients, and measurement method of quantitative values and patient characteristics [16, 1713, 14]. Therefore, quantitative values are not directly comparable between different institutions without the use of certain reference values, and each institution needs to must define reference values for standardization. In principle, such these reference values would require high reproducibility of for quantitative measurements.

Various pulse sequences, including IR[17], modified look-locker inversion recovery (MOLLI)[2], and VFA[18], have been used for measuring qT1. Of these pulse sequences, IR is most frequently used as the gold standard for qT1 evaluation. However, acquisition with IR requires considerable time (from 30 minutes to several hours). Acquisitions with MOLLI and VFA are shorter compared with IR, although MOLLI has the disadvantage of data of only one slice being obtained per acquisition, and thus, acquiring many slices is still time-consuming. By contrast, the VFA method requires a shorter acquisition time because multiple slices are acquired per acquisition.

A robust, reproducible, and standardized method is desirable for quantitative measurements. QT1 values sometimes vary, which can depend based on the location, shape, and area of the region of interest (ROI) when the ROI is set manually set. Heye et al. reported revealed that a semiautomatic ROI placement improved enhanced the reproducibility of pharmacokinetic parameters, but; however, the method is only available using dynamic enhanced MRI [1915]. Chapiro et al. highlighted emphasized the usefulness of semiautomated three-dimensional (3D) tumor segmentation but noted the necessity of acquiring contrast-enhanced T1-weighted images [2016].

Gadolinium contrast agents are commonly used in clinical practice. However, they have been known to sometimes cause side effects, making them unsuitable for patients with severe kidney dysfunction due to avoid nephrogenic systemic fibrosis[21, 22]. Therefore, contrast agents should only be used if necessary.

A highly Highly reproducible ROI placement without the need for a contrast agent is preferable for quantitative MRI.

Therefore, we developed a semiautomatic myometrial ROI placement method without a contrast agent. We hypothesized that a semiautomatic method that uses, using a 3D anatomical relationship between the myometrium and endometrium and continuity of MRI, may be useful to minimize for minimizing variation and improve the by manual ROI placements and improving ROI placement reproducibility of the ROI settings. We developed a semiautomatic myometrial ROI placement method that requires no contrast agent. The objective of this. This study is aimed to investigate the inter- and intraobserver reproducibility of qT1 measurements using manual and semiautomatic ROI settings placements.

<Discussion P17 line305>

In conclusion, the semiautomatic ROI placement algorithm had high reproducibility of qT1 measurements.

↓

In conclusion, the semiautomatic ROI placement algorithm had demonstrated high reproducibility of qT1 measurements compared with manual methods. Semiautomatic ROI placement may be useful for evaluating uterine qT1 with high reproducibility.
